# *SMC5* Plays Independent Roles in Congenital Heart Disease and Neurodevelopmental Disability

**DOI:** 10.3390/ijms25010430

**Published:** 2023-12-28

**Authors:** Matthew P. O’Brien, Marina V. Pryzhkova, Evelyn M. R. Lake, Francesca Mandino, Xilin Shen, Ruchika Karnik, Alisa Atkins, Michelle J. Xu, Weizhen Ji, Monica Konstantino, Martina Brueckner, Laura R. Ment, Mustafa K. Khokha, Philip W. Jordan

**Affiliations:** 1 Department of Pediatrics, Yale University School of Medicine, 333 Cedar Street, New Haven, CT 06510, USA; 2 Biochemistry and Molecular Biology Department, Johns Hopkins University Bloomberg School of Public Health, 615 N Wolfe St, Baltimore, MD 21205, USA; 3 Department of Biochemistry and Molecular Biology, Uniformed Services, University of the Health Sciences, 4301 Jones Bridge Rd, Bethesda, MD 20814, USA; 4 Department of Radiology and Biomedical Imaging, Yale University School of Medicine, 333 Cedar Street, New Haven, CT 06510, USA; 5 Pediatric Genomics Discovery Program, Yale University School of Medicine, 333 Cedar Street, New Haven, CT 06510, USA; 6 Department of Genetics, Yale University School of Medicine, 333 Cedar Street, New Haven, CT 06510, USA; 7 Department of Neurology, Yale University School of Medicine, 333 Cedar Street, New Haven, CT 06510, USA

**Keywords:** congenital heart disease, neurodevelopment, functional MRI, functional connectivity, hypoplastic left heart syndrome, cardiomyocytes, structural maintenance of chromosomes

## Abstract

Up to 50% of patients with severe congenital heart disease (CHD) develop life-altering neurodevelopmental disability (NDD). It has been presumed that NDD arises in CHD cases because of hypoxia before, during, or after cardiac surgery. Recent studies detected an enrichment in de novo mutations in CHD and NDD, as well as significant overlap between CHD and NDD candidate genes. However, there is limited evidence demonstrating that genes causing CHD can produce NDD independent of hypoxia. A patient with hypoplastic left heart syndrome and gross motor delay presented with a de novo mutation in *SMC5*. Modeling mutation of *smc5* in *Xenopus tropicalis* embryos resulted in reduced heart size, decreased brain length, and disrupted *pax6* patterning. To evaluate the cardiac development, we induced the conditional knockout (cKO) of *Smc5* in mouse cardiomyocytes, which led to the depletion of mature cardiomyocytes and abnormal contractility. To test a role for *Smc5* specifically in the brain, we induced cKO in the mouse central nervous system, which resulted in decreased brain volume, and diminished connectivity between areas related to motor function but did not affect vascular or brain ventricular volume. We propose that genetic factors, rather than hypoxia alone, can contribute when NDD and CHD cases occur concurrently.

## 1. Introduction

Congenital heart disease (CHD) affects 9 in every 1000 newborns [1], and while severe forms of CHD such as hypoplastic left heart syndrome (HLHS) and single ventricle disease are rare [2], they are fatal without surgical repair. In addition to the inherent morbidity of CHD, newborns with CHD are at an increased risk of extra-cardiac developmental abnormalities [3]. Up to 50% of patients with severe CHD will be diagnosed with a form of neurodevelopmental disability (NDD) [4], with 29% displaying moderate to severe developmental impairment [5]. Furthermore, 11% of adolescents meet the criteria for intellectual disability and 65% receive remedial academic or behavioral services [5].

Until recently, research has focused on the impact of hypoxia in the perinatal and perioperative periods after CHD repair as the major cause of brain injury and poor neurodevelopmental outcomes [6,7,8]. Neonates with severe forms of CHD such as HLHS are known to have a smaller head circumference at birth [9]. These complications have been attributed to reduced oxygen supply to the developing brain secondary to compromised cerebral artery oxygenation [10,11]. Attempts to act upon this hypothesis by repairing CHD in the fetal period have not improved neurodevelopmental outcomes [12], and preoperative rates of brain white matter injury have remained steady over the past 20 years despite improvements in rates of postoperative brain injury [13]. This suggests that factors outside of hypoxia may underlie the etiology of NDD in CHD patients.

Advances in genomics have enabled the identification of genetic variants as substantial contributors to the etiology of congenital cardiac disease and neurodevelopmental disorder. Genetic variants are present in one-third of all patients with CHD, with 10% of cases attributable to de novo variants [14]. Furthermore, when familial CHD patients are born with extra-cardiac congenital abnormalities, a genetic etiology has been identified in 46% of patients [15].

A genetic link between cardiac and brain development was established by showing that de novo loss of function (LOF) variants in genes expressed in the brain and heart occurred in a higher incidence in patients with CHD and NDD than in patients with CHD alone [16]. This genetic etiology was further strengthened in a recent study showing that CHD patients carried a higher burden of de novo variants in genes required for the development of the connectome, or neural functional networks of the brain [17]. This suggests that NDD in CHD patients may be caused by genetic factors that contribute to both cardiac patterning and brain connectivity. Given the high mortality rate of complex CHD and the increased risk of survivors developing a devastating neurological disease, understanding the processes that underlie these diseases could prove life-changing in terms of treatment and prognosis.

In this study, we describe a female patient diagnosed with HLHS, composed of a hypoplastic left ventricle, mitral valve hypoplasia, and a hypoplastic aorta, who was later diagnosed with a gross motor developmental delay. Subsequent whole exome sequencing of the proband and parents (trio) identified a novel de novo heterozygous 1-base pair deletion variant (c.1710del; p.(Phe570Leufs*7)) in the structural maintenance of chromosomes 5 (*SMC5*) gene (NM_015110.3), which encodes a core component of the SMC5/6 complex. This frameshift variant occurred in exon 13, in the middle of *SMC5*, and was predicted to cause premature termination that could lead to nonsense-mediated mRNA decay. The frameshift would also interrupt the flexible hinge domain of SMC5 (UniProt: Q8IY18), which connects the large intramolecular coiled coil regions and allows a crucial interaction with SMC6 to form a heterodimer. The SMC5/6 complex is a member of the SMC complex family that also comprises cohesin and condensin, which are well known for their roles in sister chromatid cohesion [18] and chromosome condensation [19], respectively. SMC5/6 is vital for maintaining chromosome structure and stability in mitosis by ensuring replication fork stability [20] and homologous recombination repair [21]. A recent report of 11 patients with variants in *SMC5* and *SLF2* (SMC5-SMC6 complex localization factor 2) displayed microcephaly, anemia, short stature, and cardiac defects [22]. Finally, *Smc5* was recently shown to be critical in neurodevelopment, as depletion of *Smc5* in the developing mouse brain impairs mitosis of neural progenitors due to abnormal chromosome segregation [20].

Following our discovery of an *SMC5* LOF variant in the proband, we created frog and mouse models. While certain de novo variants may cause a gain of function, the variant in the proband was predicted to cause non-sense medicated mRNA decay and loss of function of SMC5, so we pursued the knockout animal models. In *X. tropicalis*, *smc5* knockout (KO) reduces heart and brain size, in addition to disrupting neural progenitor cell patterning in the embryonic brain. In the mouse, null mutants of SMC5/6 complex components are embryonically lethal [23,24]. However, we demonstrate that conditional KO (cKO) of *Smc5* restricted to the central nervous system (CNS) in mice induces microcephaly and affects several neural functional networks as measured by functional magnetic resonance imaging (fMRI). Notably, these neurological changes with loss of *Smc5* in mice are observed without targeting cardiac development, suggesting that loss of *SMC5* can lead to neurodevelopmental defects independent of CHD-related hypoxia. Additionally, we show that the tamoxifen-induced *Smc5* cKO in mouse embryonic stem cell (mESC)-derived cardiac progenitors leads to depletion of mature cardiomyocytes and abnormal contractility, further indicating that CHD and NDD are occurring independently as a consequence of the *SMC5* mutation.

## 2. Results

### 2.1. Identifying SMC5 as a Candidate Genetic Etiology for NDD and CHD

**Figure 1:**
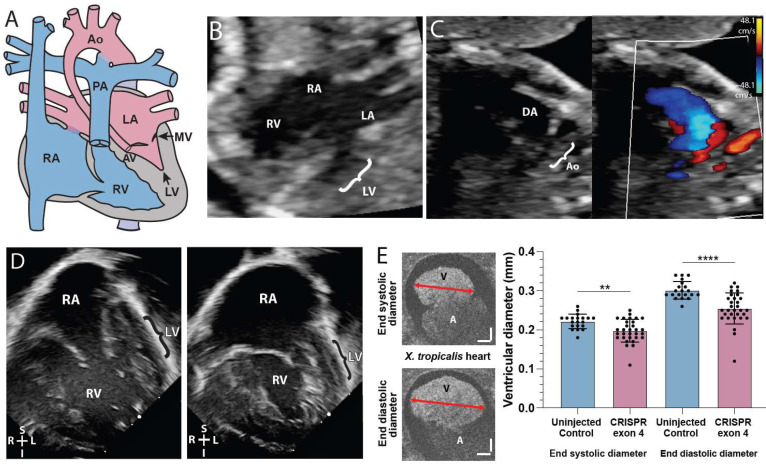
*SMC5* variant associated with impaired cardiac development in proband and frog knockdown model. (**A**) Graphic of hypoplastic left heart syndrome showing the small left ventricle (LV), small stenotic mitral valve (MV), aortic valve (AV), and hypoplastic aorta (Ao). (**B**) Fetal echocardiogram at 19 weeks gestation in an apical 4-chamber view demonstrates reduced left ventricular (LV) size compared to the right ventricle (RV) as well as the larger right atrium (RA) and small left atrium (LA). (**C**) A fetal echocardiogram at 23 weeks gestation in a view of the aortic arch demonstrates a small aortic arch (Ao) and larger ductus arteriosus (DA) with color Doppler imaging showing minimal blood flow (red) through the hypoplastic aortic arch and a much larger volume of blood flow (blue) across ductus arteriosus. Color scales estimate fluid velocity, with each color representing fluid flow toward (red) or away (blue) from the ultrasound probe located at the top of the image. (**D**) Postnatal echocardiogram at 28 days old in an apical 4-chamber view demonstrates a large right atrium (RA) and right ventricle (RV) with a thin hypoplastic left ventricle (LV). (**E**) Cross section of tadpole atria (A) and ventricle (V) of stage 45 live *X. tropicalis* embryos (n = 50). Scale bars indicating 50 μm. ** *p* < 0.01, **** *p* < 0.0001 by *t*-test in (**E**).

Fetal echocardiogram of the proband at 19 and 23 weeks of gestation and postnatal echocardiogram revealed HLHS (Figure 1A–D). HLHS is a disease caused by the under-development of the left-sided cardiac structures (mitral valve, left ventricle, aortic valve, and aortic arch) such that the left ventricular size is inadequate to maintain a sufficient cardiac output to the body (Figure 1A). Hypoplastic left heart structures can be evaluated via echocardiography in the fetal and postnatal setting and HLHS can be diagnosed if the annulus of the mitral or aortic valve has a z-score less than −2 in the setting of left ventricular hypoplasia. The patient’s prenatal anatomy indicated a significantly diminished left ventricular size (Figure 1B), with the left ventricle measuring 0.115 mL and a z-score of −4.24. These measurements suggested severe ventricular hypoplasia compared to age-matched fetal patients. The prenatal echocardiography also showed severe mitral stenosis with no sign of blood flow from the left atrium to the left ventricle (Figure 1C). In addition, there was severe aortic valve stenosis (0.2 cm, z = −4) and ascending aorta hypoplasia (0.19 cm, z = −4). Postnatal echocardiography confirmed the diagnosis of HLHS and the mitral valve was found to be atretic with no flow across it. Due to a lack of blood flow into the left ventricle, the left ventricle became increasingly diminutive in comparison to the right ventricle and can be visualized as a small thin blind-ended pouch (Figure 1D). The patient underwent initial palliative repair with aortic arch reconstruction (Norwood with Damus-Kaye Stansel procedure) [25] and Sano shunt placement after birth [26]. At age 6 months, repair of the partial anomalous pulmonary venous return (left lower pulmonary vein to left atrial appendage) and bidirectional Glenn procedures were performed with a takedown of the Sano shunt. The patient’s pediatrician identified a gross motor delay at age 9 months, namely due to a delay in crawling and transitioning between movements and positions while prone. The patient required 3 months of weekly physical therapy.

Given evidence of HLHS and gross motor delay, the proband and parents underwent genetic testing to identify candidate genetic etiologies. Genetic sequencing identified three rare homozygous recessive variants (*SMC5*, *TRPA1,* and *SPANXN2*), three rare de novo variants, and 6 compound heterozygous variants (minor allele frequency < 0.01) in the patient (Supplementary Table S1). As de novo variants play an important role in the development of CHD and NDD [16], we focused on the de novo variants in genes *SMC5* and *TPRA1*. We did not assess *SPANXN2* as it is primarily expressed in the testis and is classified as a sperm-specific protein with a role in testicular cell tumors [27]. To investigate further, we performed a gene KO via CRISPR-Cas 9 mediated gene editing with 2 non-overlapping sequences of *smc5* and *tpra1* in *X. tropicalis* embryos. *tpra1* KO did not show a significant level of heart defects (Supplementary Figure S1; *p* = 0.6212, n = 71). On the other hand, *smc5* KO showed a cardiac phenotype (Figure 1E). CRISPR-Cas9 targeting against *smc5* induced edits (insertions or deletions) in 87.6–89.7% of *X. tropicalis* genomic DNA samples (Supplementary Figure S2).

### 2.2. smc5 KO Reduces Cardiac Ventricle Size in X. tropicalis

We investigated the cardiac ventricular size in *X. tropicalis smc5* KOs due to the presence of an underdeveloped left ventricle in our patient. Using optical coherence tomography (OCT), we quantified cardiac ventricular size by measuring maximum ventricular diameter at the end of systole (contraction) and end of diastole (relaxation) in stage 45 tadpoles. *smc5* KO caused a 10.8 ± 3.0% reduction in end-systolic ventricular length (*p* = 0.0018 by unpaired t-test, n = 30) and a 15.5 ± 3.2% reduction in end-diastolic ventricular length (*p* = 2 × 10^−5^ by unpaired *t*-test, n = 30) indicating that ventricular size is reduced in tadpoles depleted of Smc5 (Figure 1E).

### 2.3. SMC5 Is Required for Mouse Cardiac Development and Function

**Figure 2:**
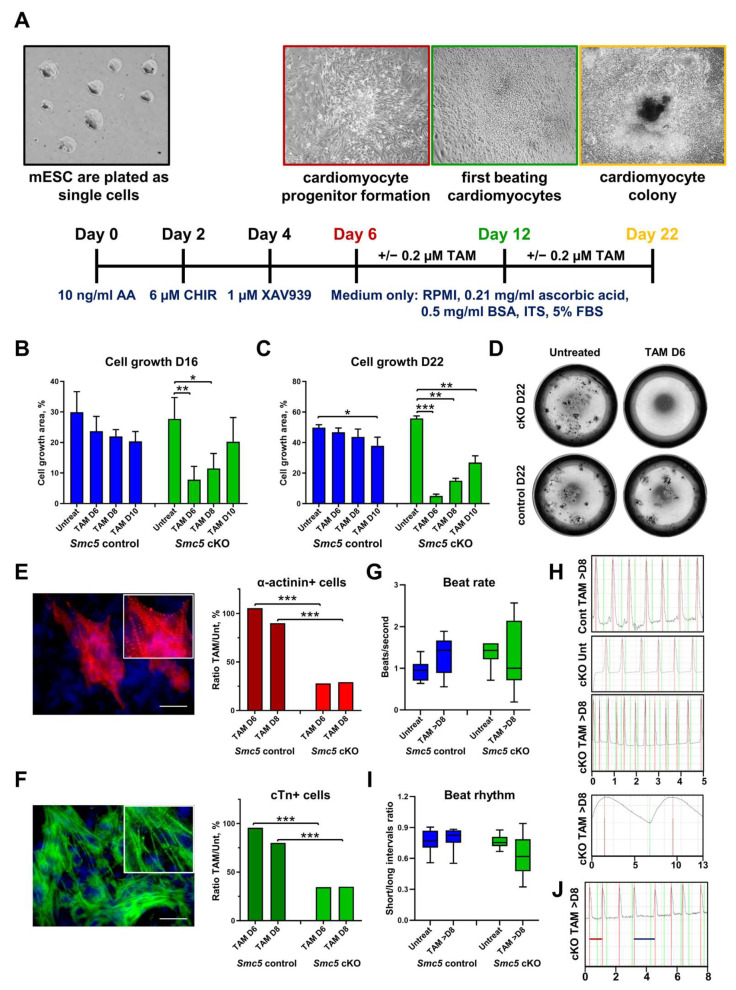
Smc5 is required for mouse cardiac development and function. (**A**) The outline of mESC cardiac differentiation and tamoxifen treatment. (**B**–**D**) *Smc5* cKO in cardiac progenitors causes a significant reduction in cell proliferation. Tamoxifen (TAM) was added on day 6, day 8, and day 10 of mESC differentiation. The cell outgrowth was evaluated on day 16 (**B**) and day 22 (**C**,**D**). Images of live cell cultures are shown for differentiation day 22 (**D**). Data are a cumulative of two independent experiments performed in replicas. Data was assessed using the Mann–Whitney *t*-test and significant comparisons are given as * *p* < 0.05, ** *p* < 0.01, and *** *p* < 0.001, and other comparisons were deemed non-significant. (**E**,**F**). *Smc5* cKO in cardiac progenitors causes a significant reduction in resulting cardiomyocytes based on the evaluation of sarcomere proteins α-actinin (red) and cardiac troponin T (cTn) (green) expression. Tamoxifen (TAM) was added on day 6 and day 8 of mESC differentiation. Cells were evaluated on day 18. The ratio of tamoxifen-treated to untreated cells is shown (n ≥ 5 × 10^3^ total cells). Examples of α-actinin (red) (**E**) and cardiac troponin T (cTn) (green) (**F**) expression in mESC-derived cardiomyocytes are shown. Scale bar 25 μm. Data was assessed using the Mann–Whitney t-test and significant comparisons were given as *** *p* < 0.001, and other comparisons were deemed non-significant. (**G**) *Smc5* cKO in cardiac progenitors results in higher variability in beat rate (n = 6–16). (**H**) Examples of TAM-treated control (Cont) (Supplementary Movie S1), untreated (Unt) cKO (Supplementary Movie S2), and TAM-treated cKO cardiomyocytes with fast and slow beat rate are shown (Supplementary Movies S3–S5) (scale, seconds). (**I**) *Smc5* cKO in cardiac progenitors results in higher variability in beat rhythm (n = 7–12). (**J**) An example of TAM-treated cKO cardiomyocytes with short (red line) and long (blue line) intervals between contractions is shown (Supplementary Movie S6) (scale, seconds).

Embryonic stem cells (ESCs) can be differentiated into diverse tissue-specific cell types and have been used for disease modeling for decades [28,29]. Therefore, we utilized tamoxifen-inducible *Smc5* cKO mESC lines that we previously generated [30]. To evaluate the requirement of Smc5 during mouse cardiac development, we induced mESC differentiation into the cardiac lineage and added tamoxifen during cardiomyocyte specification between days (D) 6 to 12 of differentiation (Figure 2A). This range was chosen to assess the effects of inducing *Smc5* cKO in cultures enriched for cardiomyocyte progenitors (D6) or cardiomyocytes (D10 to 12).

The most dramatic reduction in *Smc5* cKO cell outgrowth was observed when tamoxifen was added on day 6 (D6) of differentiation, leading to a 3.55-fold decrease by D16 and 11.24-fold decrease on D22 of differentiation (Figure 2B–D). In contrast, when tamoxifen was added to D10 of differentiation only a 1.37- and 2.06-fold decrease in cell growth was observed by D16 and D22, respectively (Figure 2B,C). These results are consistent with our previous observations that demonstrate Smc5 is critical for mESC and progenitor cell proliferation and *Smc5* cKO leads to apoptosis [20,30]. In contrast, in differentiated cells, such as mouse embryonic fibroblasts or spermatocytes, *Smc5* cKO does not result in rapid cell death but can lead to increased sensitivity to DNA damage [31,32]. Only slight reductions in cell proliferation were observed in control cells at D16 and D22 of differentiation when tamoxifen was added on D6 to D10 (Figure 2B–D).

Next, we evaluated the number of cardiomyocytes in cell cultures by evaluating the expression of cardiomyocyte-specific sarcomere proteins α-actinin and cardiac troponin T (cTn) (Figure 2E,F) [33,34]. A 3.78- and 3.09-fold reduction in α-actinin+ cells was observed in the *Smc5* cKO compared to the control cell line, when tamoxifen was added to D6 and D8 of differentiation, respectively (Figure 2E). Similarly, a 2.76- and 2.29-fold reduction in cTn+ cells was observed in the cKO sample compared to the control (Figure 2F). These data demonstrate that Smc5 is important for cardiomyocyte differentiation.

We then analyzed whether *Smc5* cKO can affect cardiomyocyte functionality by assessing beat rate and beat rhythm (Figure 2G–J and Supplementary Movies S1–S6). Spontaneously contracting cardiomyocyte clusters were not formed if tamoxifen was added on D6 of differentiation, which is expected considering the importance of Smc5 function for the viability of cardiomyocyte progenitors (Figure 2B–D). If tamoxifen was added at later stages of differentiation (D8 or D10), *Smc5* cKO samples demonstrated higher variability in beat rate (0.19–2.57 beats/second for *Smc5* cKO versus 0.56–1.89 beats/second for the control) and greater contraction arrhythmia (0.32–0.94 beat interval ratio for *Smc5* cKO versus 0.55–0.90 beat interval ratio for the control) compared to untreated or control samples (Figure 2G–J and Supplementary Movies S1–S6).

These experiments, in addition to results obtained using *X. tropicalis smc5* Kos, support the hypothesis that SMC5 is necessary for normal heart development and function in humans.

### 2.4. Smc5 Is Required for Proper Brain Size in X. tropicalis

**Figure 3:**
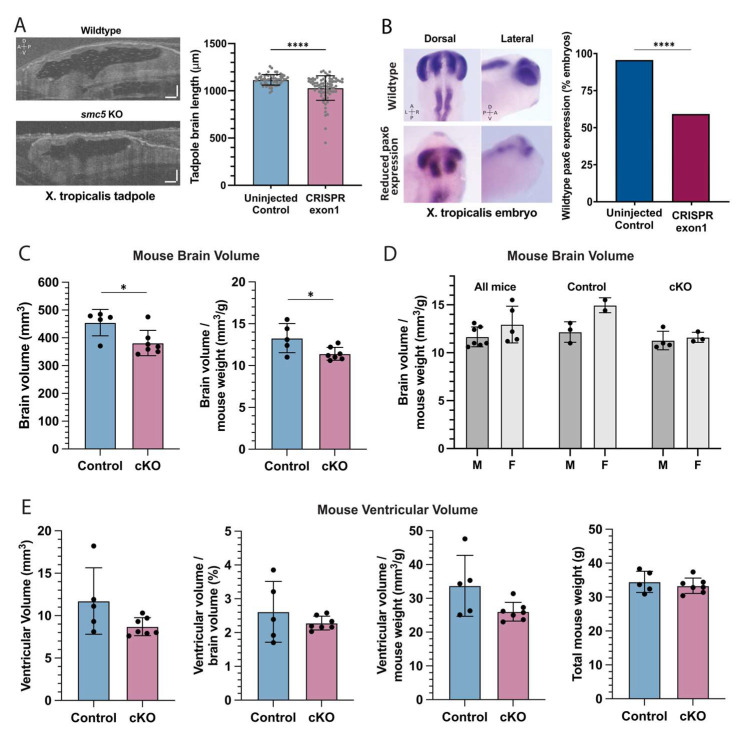
*smc5* knockout alters brain development in frogs and mice (**A**) Sagittal cross-section of stage 45 *X. tropicalis* brain in control and *smc5* KO (CRISPR exon 1) embryos. Scale bars presenting 100 μm. (**B**) Whole-mount in situ hybridization of *pax6* in stage 28 *X. tropicalis* embryos, highlighting reduced expression in the *smc5* KO (CRISPR exon1) brain, notochord, and developing eye. Images show *pax6* distribution rather than scale. (**C**) Brain volume and brain volume normalized by weight in control and *Smc5* cKO mice measured by MRI. (**D**) Brain volume normalized by weight compared between male (M) and female (F) mice, showing all, control, or *Smc5* cKO mice. (**E**) Brain ventricular volume of control or *Smc5* cKO mice is shown as raw volume, ventricular volume normalized by brain volume, or ventricular volume normalized by weight. Legend: left (L), right (R), dorsal (D), ventral (V), anterior (A) and posterior (P), * *p* < 0.05, **** *p* < 0.0001 by *t*-test in (**A**,**C**–**E**) and by chi-square analysis in (**B**).

The proband had both HLHS and signs of NDD as demonstrated by gross motor delay. While developmental delays are common in the setting of CHD, microcephaly frequently occurs with severe CHD [35]. Having established reduced cardiac ventricular size in our *smc5* KO frog model (Figure 1D), we next investigated brain length in *X. tropicalis* using OCT in stage 45 embryos. Brain length was used as a proxy for brain size given the limitations of volumetric measurements via OCT at the time of data collection. Embryos targeted for *smc5* KO had a 7.8 ± 1.6 % decrease in brain length (*p* = 4 × 10^−10^ by *t*-test, n = 78; Figure 3A). Brain growth and structural development depend on neural progenitor expansion, differentiation, and migration into corresponding cortical layers [36]. Neural progenitor cells are characterized by the expression of the *pax6* transcription factor [37], so we investigated *pax6* expression in the developing *X. tropicalis* brain. The *smc5* KO in *X. tropicalis* embryos reduced *pax6* expression in 41% of embryos at stage 28 (*p* = 9.7 × 10^−5^, n = 51; Figure 3B), indicating that neural stem cell proliferation was impaired with depletion of Smc5.

We observed altered brain development when *smc5* is mutated in the developing tadpole. However, due to the reduced cardiac ventricular size, one could propose that the observed neurological changes may be secondary to reduced cardiac output and impaired oxygen delivery to the embryonic brain. Given that variants in the SMC complex proteins have been associated with both CHD and NDD [38,39,40,41], we hypothesized that *SMC5* may play a role in neural development that is independent of cardiac function. To test this directly, we pursued a CNS-specific *Smc5* KO mouse model.

### 2.5. Reduced Brain Size in Smc5 cKO Mice Occurs Independent of CHD

To induce *Smc5* depletion in the mouse brain, we used a strain harboring a *Smc5*-flox allele and the *Nestin*-*Cre* transgene [20]. Nestin expression is limited to the CNS in mice [42] and *Nestin*-*Cre* mice have been previously used in studying neuropsychiatric disease [43]. The larger brain size of the mouse compared to the frog allows us to use MRI to measure brain size with sub-structure resolution and evaluate brain function in vivo. The average brain volume of *Smc5* cKO mice, at 381.4 ± 45.5 mm^3^, was smaller than that of control mice, at 475.7 ± 8.1 mm^3^ (−19.8%, *p* = 0.04 by *t*-test) (Figure 3C). This difference persisted when brain volumes were normalized by body weight (Figure 3C). For *Smc5* cKO, control, or all mice, the ratio between brain volume and body weight showed no difference between male and female mice (*p* > 0.05) (Figure 3D). Further, brain ventricular volume showed no difference between *Smc5* cKO and control mice, with or without normalization to total brain volume or body weight, underscoring that the brain volume difference is driven by changes in tissue, not the fluid compartment (Figure 3E).

**Figure 4:**
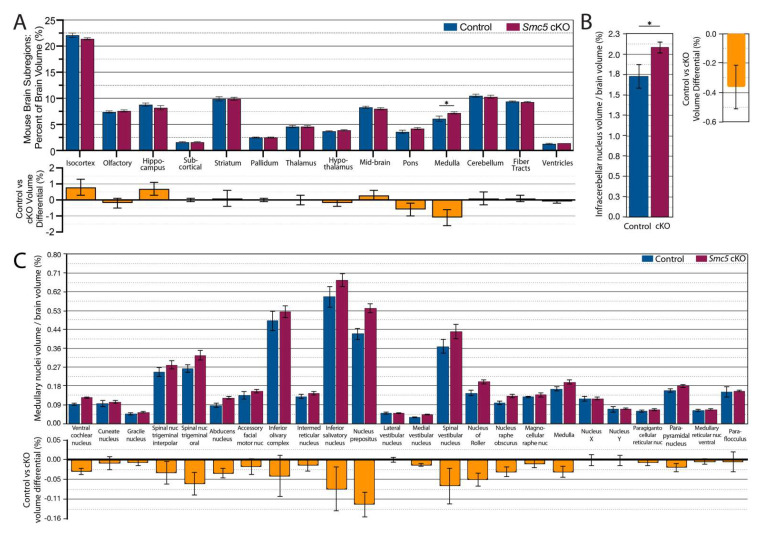
*Smc5* cKO reduces brain volume in most regions except medulla. (**A**) Percentage of subregion volume compared to total brain volume of control vs *smc5* cKO mice. A significant increase in medulla size is noted with *Smc5* cKO. (**B**) The volume of the medullary infracerebellar nucleus is significantly (*p* = 0.03) enlarged in *Smc5* cKO animals as normalized to total brain volume. (**C**) The medulla is uniformly enlarged across all remaining medulla subregions as demonstrated by medulla volume normalized to total brain volume; however, we see no significant differences between groups. Nuc.: nucleus, * *p* < 0.05, n = 12 for all experiments.

We next asked if the reduction in brain volume acted globally across the brain or was region-specific. In the BioImageSuite (BIS) toolkit, there are three versions of the mouse Allen Brain Atlas at successively more fine-grained resolutions [44]. We began a regional brain volume comparison between *Smc5* cKO and control mice with the coarsest version of the atlas (containing 14 regions per hemisphere). Qualitatively, reduction in volume with *Smc5* cKO was broadly distributed across the isocortex, hippocampus, mid-brain, and cerebellum (Figure 4A). Individually, these regions show only small differences (<1% of the total brain volume). On the other hand, the medulla in *Smc5* cKO mice is larger than in control mice (*p* < 0.05 by *t*-test). Given the role of the medulla in directing input around balance and gross motor control, as well as its role in the autonomic nervous system and regulation of heartbeat, we investigated the substructures of the medulla to see if some were more significantly impacted by loss of *Smc5*. At the medium-scale resolution (containing 160 regions per hemisphere), the medulla is broken down into 25 sub-regions. We observed that the increased size of the medulla in *Smc5* cKO mice is uniformly distributed, with a significant increase in the size of the infracerebellar nucleus (Figure 4B), as well as uniform enlargement across sub-divisions (Figure 4C). While not significantly enlarged, larger regions of the medulla such as the nucleus prepositus, inferior salivatory nucleus, and spinal vestibular nucleus show a greater proportion of enlargement compared to other regions.

In summary, the changes in brain volume observed for the *Smc5* cKO, can result in functional connectivity differences, which can lead to cognitive impairments and motor sensory anomalies, the latter of which we previously confirmed for this model [20].

### 2.6. Smc5 cKO Does Not Significantly Impact Brain Vascular Volume or Spatial Distribution

**Figure 5:**
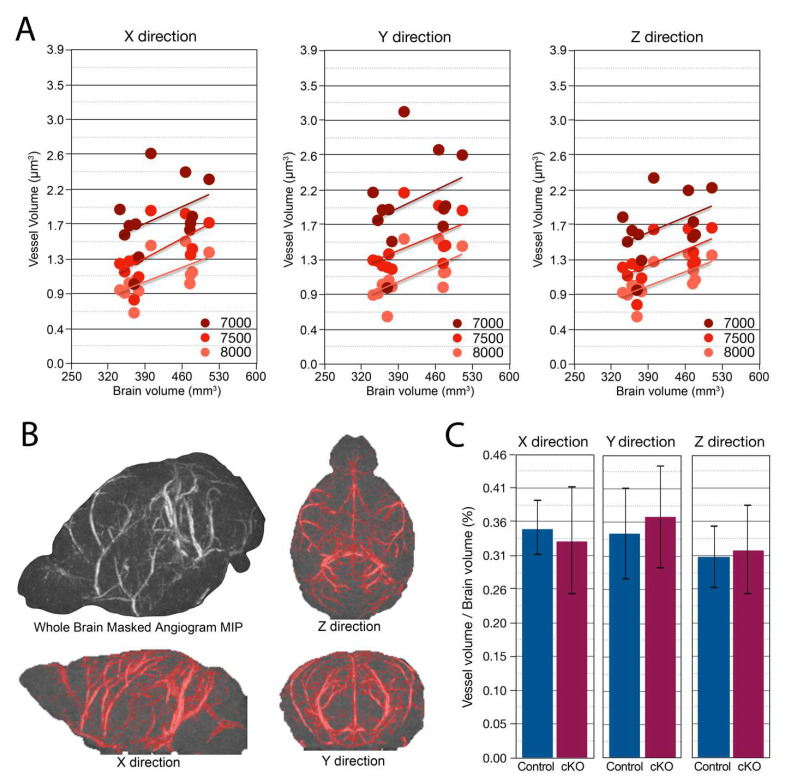
Brain vascular volume and spatial distribution are preserved with *Smc5* cKO. (**A**) Maximum Intensity projection (MIP) of vascular volume across multiple signal intensity thresholds (7000, 7500, 8000) and direction (X, Y, & Z) showing increasing vessel volume correlated with increasing brain volume (n = 12). (**B**) Example of Masked Angiogram MIP of a control mouse (n = 1). Red shading shows masked (by signal intensity threshold) angiogram in X, Y, and Z directions. The threshold shown is 7500 (arbitrary signal intensity units). (**C**) Vascular volume normalized to total brain volume in X, Y, & Z directions has no significant difference between control and cKO mice (n = 12).

Disrupted angiogenesis in the developing brain or altered vascular development can lead to global or regional hypoxia. Therefore, we investigated gross vascular volume and distribution via MRI to evaluate whether *Smc5* cKO indirectly disrupted large blood vessel growth, which could potentially contribute to the observed differences in brain size. High, medium, and low signal intensity thresholds were chosen by visual inspection of the time-of-flight angiography data and held constant across mice. In each direction, and at each signal intensity threshold, vessel volume was correlated with total brain volume (R^2^ = 0.29 ± 0.12, mean ± SD) (Figure 5A). Vascular volume was computed using a maximum intensity projection in each of three directions using three signal intensity thresholds (Figure 5B). With or without normalizing to total brain volume, no differences were found between *Smc5 cKO* and control mice in intravascular volume (Figure 5C).

### 2.7. Smc5 cKO Disrupts Functional Connectivity in the Mouse Brain

Neurological development requires proper communication between brain structures through neural networks. The sum of all functional connections within the brain, known as the connectome, can be assessed using blood-oxygen-level-dependent (BOLD) fMRI [45]. The connectome is comprised of connectivity strengths which are defined as the correlation of the BOLD signal between all pairs of brain regions. Region-to-region connections are called edges, and a collection of edges with a common function is called a network. Using the coarse-scale Allen Atlas (14 regions per hemisphere), we computed a connectome for each mouse (Supplementary Figure S3). In comparing connectomes across all mice, any two connectomes from two different mice, either both male, both female, or one male and one female, showed moderate similarity (R^2^ = 0.27, R^2^ = 0.30, and R^2^ = 0.26, respectively). We observed no gross differences between the groups in connectome edge strength when comparing both inter- and intra-hemisphere, as shown by normal distributions of control and cKO histograms (Supplementary Figure S4A).

To isolate identifying connectivity patterns of interest, we identified edges that differentiate mice within the group (control vs. control or cKO vs. cKO, subject-specific) as well as edges that can differentiate control mice from *Smc5* cKO mice (control vs. cKO, group-specific) (Supplementary Figure S4B). Each mouse was compared to other mice within the group and across groups, and the connectome of each mouse was subtracted from the connectome of another, resulting in difference matrices; difference matrices were moderately, but significantly correlated (control R^2^ = 0.11, cKO R^2^ = 0.20, between groups R^2^ = 0.21, *p* < 0.00005; Supplementary Figure S4C). Within each group (control vs. control or cKO vs. cKO), as well as between groups (control vs. cKO), we found distinguishing edges that were reproducible across pairs of mice. Edges that distinguish controls from one another also distinguish *Smc5* cKO mice from one another (control vs. control compared to cKO vs. cKO, R^2^ = 0.24, *p*~0). Further, edges that distinguish controls from one another are negatively correlated to edges that distinguish controls from *Smc5* cKO mice (control vs. control compared to control vs. cKO, R^2^ = 0.40, *p*~0). On the contrary, edges that distinguish *Smc5* cKO mice from one another show no relationship to edges that distinguish control from *Smc5* cKO mice (cKO vs. cKO compared to control vs. cKO, *p* > 0.5). Together, these results indicate that there is a subject-specific connectivity pattern shared across each group that differentiates control mice from each other and differentiates *Smc5* cKO mice from each other. In addition, a separate connectivity pattern distinguishes control from *Smc5* cKO mice.

**Figure 6:**
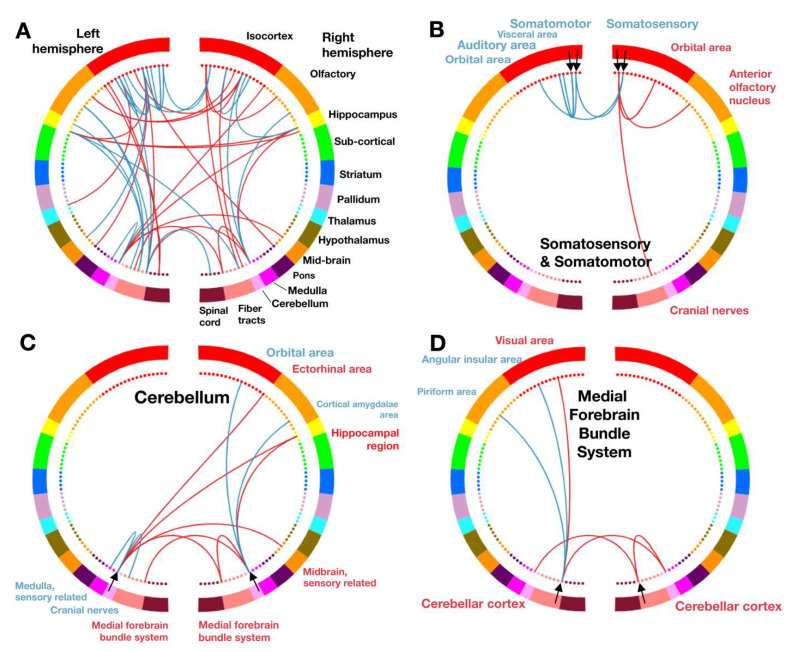
*Smc5* cKO disrupts somatomotor/somatosensory, cerebellar, and medial forebrain functional connectivity. Connectivity maps use each color in a semicircle to represent a different brain region, with the left and right semicircle representing each respective mouse brain hemisphere. While connections exist between all regions, lines shown between regions of interest (ROI, black arrow) denote a change in connectivity that distinguishes between control and *Smc5* cKO mice. Red lines represent an increase in synchrony between ROIs (red text) and blue lines represent a decrease in synchrony between ROIs (blue text). (**A**) Combined connectivity changes across the brain, with all ROIs shown. (**B**–**D**) Connectivity patterns associated with each interrogated ROI (named by black text) are individually shown. (**B**) Somatosensory and somatomotor connectivity maps, with somatomotor ROI represented by innermost black arrows, and somatosensory ROI represented by outermost black arrows. (**C**) Cerebellar connectivity map and (**D**) Medial forebrain bundle system connectivity map. n = 12 for all experiments.

To examine which brain regions are implicated in distinguishing *Smc5* cKO from control mice, we binarized the connectivity pattern (R^2^ > 0.2) to create a network (Supplementary Figure S4D). At the coarsest scale, the Allen Atlas regions including the olfactory cortex, isocortex, hippocampus, medulla, pons, and cerebellum were included in the network (Supplementary Figure S4D). We repeated our analyses at the medium-scale Allen Atlas resolution (Figure 6A) and analyzed connectivity patterns between each region of interest (ROI) studied (Supplementary Figure S5). Notably, the changes in the somatosensory and somatomotor connectivity are reflected in mouse behavioral tests performed in our study using the same *Smc5*-flox *Nestin*-Cre mouse strain [20]. Specifically, the delayed responsiveness to an adhesive patch and decreased ability to hang on a wire mesh screen seen in mice is potentially due to the decreased interhemispheric connectivity between left somatosensory and right somatomotor cortices [20] (Figure 6B). Additionally, mice displayed decreased exploratory behaviors in cylinder tests which could be due to abnormal connectivity patterns between the medial forebrain bundle and the cerebellum [20] (Figure 6C,D).

## 3. Discussion

### 3.1. Cardiac Phenotype Associated with SMC5 Variants Informs the Pathophysiology of HLHS 

The molecular pathophysiology of HLHS remains poorly understood, and there is no common genetic variant among HLHS patients [46]. While multiple candidate HLHS genes have been proposed by whole exome sequencing (*GJA1*, *HEY2*, *PTCH1*, *IRX4*, *BMP2*, *MYH6*), the prevalence of variants in these genes in the HLHS population is low and they have not been replicated in animal models [29,46]. While some forms of CHD such as heterotaxy are caused by single gene variants [47], the low prevalence of HLHS combined with a high rate of clinically insignificant polymorphisms in the general population make detecting a monogenic etiology difficult.

Variants in *SAP130* and variants in the *PCDHA* gene family have been detected in HLHS patients. In mouse and zebrafish models, *Pcdha9* mutations lead to valvular defects, whereas *Sap130* mutations primarily affect cardiac ventricular development [48]. Similarly, KO of *smc5* in *X. tropicalis* most affects ventricular growth, suggesting that in some patients multiple genetic variants may underlie the complex left-sided heart defects seen in HLHS. However, new work details how KO of *RBFOX2* orthologs in zebrafish (*rbfox2* and *rbfox1l)* decrease the cardiac ventricular size, cause outflow tract hypoplasia, and decrease cardiomyocyte contractility [49], indicating both monogenic and polygenic variants may play a role in the etiology of HLHS.

In our study, we identify *SMC5* as a candidate gene from a HLHS patient. *SMC5* variants have been identified in patients with mild forms of CHD (persistent ductus arteriosus, arterial septal defect, or mild supravalvular pulmonic stenosis), all of which resolved without intervention [22]. Here, we report the association of a variant in *SMC5* with hypoplastic left heart syndrome, the first association of *SMC5* with a cyanotic heart lesion (Figure 1). Within, we demonstrated that *smc5* mutation resulted in reduced cardiac ventricular size in a frog animal model (Figure 1). In addition, our experiments with *Smc5* cKO during the specification of mouse cardiac lineage clearly demonstrate the necessity of Smc5 for the expansion of cardiac progenitors and their differentiation into functional cardiomyocytes (Figure 2).

### 3.2. SMC5 Is Required for Cardiac Development

Our understanding of the role of the SMC5/6 complex in cardiac development continues to expand. The first identified patient with cardiac disease carried a variant in *NSMCE2*, a component of the SMC5/6 complex, which causes primordial dwarfism with microcephaly [50]. This patient was reported to die at age 33 from a sudden cardiovascular event [50]. Recent work on *NSMCE2* and *SMC5* demonstrates that the p.Arg372del variant in *SMC5* can destabilize the interaction of SMC5 and NSCME2 [51], indicating that *SMC5* variants can potentially play a role in heart disease by disrupting its interaction with *NSMCE2*. Additional work has described patients with variants in *SMC5* and a variety of non-cyanotic cardiac defects, including persistent ductus arteriosus, atrial septal defect, or supravalvular pulmonic stenosis [22]. The variety of structural defects in these patients involves different pathways in cardiac development, suggesting that the resulting cardiac defects may be secondary to which cell types are most affected during cardiac development.

Embryonic cardiac development requires the migration and proliferation of different cell types during critical periods [46], and may be disrupted by impaired differentiation of embryonic structures. The proband in our study presented with the most complex cardiac anatomy associated with variants in *SMC5*, namely severe mitral stenosis, aortic valve stenosis, ascending aorta hypoplasia, severely diminished left ventricular size, and partial anomalous pulmonary venous return. Given the evidence that KO of *smc5* in zebrafish and mice leads to defects in DNA repair and disrupts cell cycle progression in the brain [20,22], we hypothesize that disrupted proliferation of embryonic heart progenitor cell populations is similarly affected, resulting in underdeveloped hearts. This hypothesis is supported by findings of increased apoptosis and signs of cell cycle arrest in mice with HLHS secondary to mutations in *Sap130* and *Pcdha9* [46]. Our experiments with *Smc5* cKO during the specification of mouse cardiac lineage clearly demonstrate the necessity of Smc5 for the expansion of cardiac progenitors and their differentiation into functional cardiomyocytes (Figure 2).

### 3.3. SMC5 Is Required for Development of Proper Brain Structures and Function

The three known SMC complexes (SMC1/3, SMC2/4, and SMC5/6) function in diverse roles in DNA replication and repair, but all have been associated with microcephaly due to abnormal neurodevelopment [20,22,40,41,50,51,52,53]. Prior work on the SMC5/6 complex demonstrates increased DNA damage in the mouse brain in a *Smc5*^K371del^ knock-in mouse model [51], as well as microcephaly and increased apoptosis in the zebrafish brain with *smc5* KO [22]. Further work with *Smc5* cKO in the CNS shows that defects in brain growth with loss of SMC5 are secondary to DNA bridges and chromosomal missegregation in neural progenitor cells, subsequently leading to brain microcephaly and reduced cortical size [20]. Our work builds on these findings, confirming not only a microcephalic phenotype and reduced cortical size but also a global reduction in brain structures (Figure 4). Additionally, *smc5* KO in the frog supports the hypothesis of impaired replication and differentiation of neural progenitor cells, as shown by abnormal expression and patterning of neural progenitor marker *pax6* (Figure 3).

MRI of *Smc5* cKO animals demonstrates that all regions of the mouse brain are uniformly reduced in size, except for the medulla which is enlarged compared to control animals. The broad impact on brain structures is reflected by the abnormal FC seen across cortical, subcortical, and brain stem regions of the CNS in *Smc5* cKO mice. Given the sensorimotor deficits found in this *Smc5*-flox *Nestin*-Cre strain [20], we were particularly interested in finding reduced interhemispheric connectivity between the somatomotor and somatosensory regions in *Smc5* cKO animals, as well as the decreased connectivity between the cerebellum and the medulla (Figure 6). This finding was more curious in the context of enlargement of the medulla in *Smc5* cKO animals. The medulla appears to be uniformly enlarged, though the infracerebellar nucleus and spinal vestibular nucleus are enlarged in a greater proportion compared to other medullary substructures. The spinal vestibular nucleus functions in balance and spatial navigation, and the infracerebellar nucleus is critical to the vestibulomotor system, both contributing to directionality and stability in movement. While further research is needed, the enlargement of these medulla substructures may represent compensatory mechanisms to partially overcome altered FC between the somatomotor, somatosensory, and cerebellar structures.

### 3.4. Variants in SMC5 Are Associated with Atelis Syndrome

Defects in SMC5 and the SMC5/6 complex lead to DNA replication stress and impair chromosomal segregation, triggering apoptosis by impairing cell cycle progression [20,22]. Recent work demonstrated that mutations in *SMC5* and *SLF2* disrupt the RAD18-SLF1/2-SMC5/6 pathway that is required for DNA repair, leading to chromosomal breakage and segmented chromosomes [22]. Defects in the SMC5/6 complex at the cellular level lead to direct impairment of brain growth and cortical development by causing apoptosis of neural progenitor cells [20]. In addition to reduced brain size, NDD, and CHD, patients with variants in *SLF2* or *SMC5* were found to have ocular abnormalities, growth restriction, anemia, and skin hyperpigmentation, a condition recently named Atelis syndrome [22].

Notably, the proband used in our study was not diagnosed with any vision or eye defects, anemia, or skin changes. The patient had impaired growth in length and weight since birth but was the appropriate size for gestational age at birth. Given the difference in phenotype between our patient and the Atelis syndrome phenotype, we cannot determine at this time whether this patient can be categorized with Atelis syndrome. However, in examining the phenotype of Atelis syndrome patients with *SMC5* variants, while NDD and cardiac phenotypes are common, the other signs such as eye defects, skin hyperpigmentation, or anemia are not present in all patients. This suggests a range of phenotypes may exist, dependent on which part of the RAD18-SLF2-SMC5/6 pathway is affected.

The molecular functions of the SMC5/6 complex are yet to be fully elucidated. However, there are clear indications that SMC5/6 is important for the completion of DNA replication prior to chromosome segregation [20,21,54,55,56,57]. In yeast and mammalian cells, SMC5/6 is chromatin-associated throughout interphase, with a constitutively bound population and a freely diffusing population that interacts transiently with DNA [54,58]. During mitosis, SMC5/6 appears to remain chromatin-bound but is enriched at the centromeres and pericentromeric regions as well as ribosomal DNA and telomeres [30,54,59,60,61,62]. Numerous reports have postulated a role for SMC5/6 in promoting the stability of stalled replication forks, with direct implications for preventing fork collapse and unscheduled recombination [55,56,57]. Several questions remain regarding the core function of SMC5/6 that makes it such an essential guardian of the replication fork. Recent revelations into the structure and DNA-binding properties of SMC5/6 have illuminated a potential role as a molecular machine, facilitating the entrapment, compaction, and stabilization of replication-associated DNA tertiary structures [63,64]. Other studies have suggested that SMC5/6 may function as a hub of protein-protein interaction by way of its coiled-coil arms, which in notable contrast to the other SMC complexes, contain extensive binding sites for other proteins as revealed by cross-linking mass spectrometry [65]. These features are telling when viewed next to the fact that SMC5/6 is the only member of the SMC complex family to harbor two distinct components with enzymatic activities, NSMCE1 an E3 ubiquitin ligase, and NSMCE2 an E3 SUMO ligase, the targets of which are still mostly unidentified. Perhaps the broad necessity of SMC5/6 for chromosomal transactions, from DNA replication to mitosis, is reflective of its capacity to both enact structural changes, in a similar fashion to its SMC complex cousins, and to coordinate the response of other proteins at the replication fork via posttranslational modification. Further characterization of replication fork dynamics using more fine-tuned model systems and imaging techniques will be integral to further elucidating the role of SMC5/6 at the replication fork.

Beyond the roles during DNA replication and chromosome segregation in proliferating cells, the functions of SMC5/6 in post-mitotic cells are mostly unexplored. Unlike SMC5/6, it is well established that cohesin and condensin have roles in regulating transcription [66,67,68]. Therefore, the SMC5/6 complex may have roles in regulating transcription in post-mitotic neurons and cardiomyocytes. For instance, neuronal and cardiomyocyte activity triggers the formation of DNA double-strand breaks (DSBs) on promoters of early-response genes, which in turn stimulates their expression [69,70]. The DSBs that form on promoters of early response genes are mediated by Topoisomerase IIβ (TOP2B) [69,70,71,72]. Mutations of *TOP2B* lead to neurodevelopmental disorder and diastolic dysfunction [70,73,74,75,76,77,78,79], which are phenotypically similar to when SMC5/6 components are mutated. Research using budding yeast and mammalian cell culture has shown that SMC5/6 and TOP2 physically and functionally interact [30,80,81]. Therefore, it can be hypothesized that SMC5/6 mediates gene expression in conjunction with TOP2B, which is critical for the normal function of post-mitotic neurons and cardiomyocytes.

### 3.5. SMC5 Plays an Independent Role in NDD in the Setting of CHD

Infants with CHD are at greater risk of cerebral structural abnormalities as well as aberrant functional connectivity patterns [8,11,45]. A clear mechanism has been suggested for the role of hypoxia-induced NDD in CHD patients. Cyanotic or ductal-dependent CHD patients have decreased cerebral oxygenation [7,82], and hypoxia is shown to impair neural progenitor cells in the setting of CHD [8]. It is unclear if the impact of hypoxia on brain FC is greatest before or after birth; however, studies have identified abnormalities in FC networks in CHD patients before cardiac surgery [83], suggesting that alterations in FC occur early in development. Patients with HLHS are known to have smaller head sizes at birth [11,45] suggesting oxygenation has an important role in fetal brain growth [10]. In our study, we have the advantage of studying the impact of *smc5* KO on the brain with and without associated CHD. While we observe reduced brain size in the presence of CHD (Figure 3A), we also find that loss of *Smc5* reduces brain volume without the impact of CHD-induced hypoxia (Figure 3C–E and Figure 5). This suggests that SMC5 acts independently in brain and cardiac development and that NDD may arise without CHD-associated hypoxia. Other studies on NDD in CHD patients demonstrate reduced white matter volume [84] and reduced brain size [85] in patients with non-cyanotic heart lesions as well, further removing the impact of hypoxia on the development of NDD secondary to CHD. We propose that patients with CHD are at additional risk for NDD separate from the impact of cerebral oxygenation, primarily due to genetic factors that may play an independent role in the development of brain structure and connectivity.

**Figure 7:**
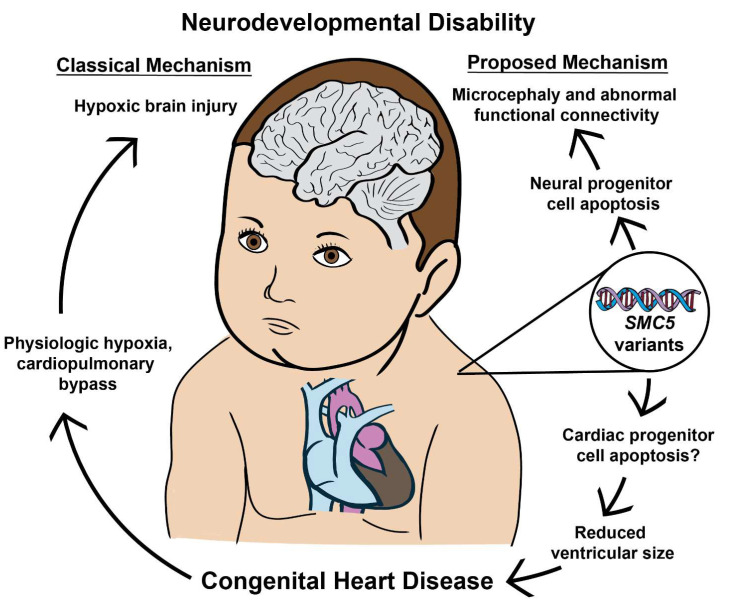
SMC5 malfunction during embryonic development produces CHD and NDD through independent processes. The data presented indicates that neurodevelopmental defects can occur with and without concurrent CHD. Furthermore, SMC5 mutations can alter brain FC and cause developmental delays.

In summary, we demonstrate that loss of SMC5 in the developing embryo produces CHD and NDD through independent processes and that microcephaly occurs with and without concurrent CHD. Furthermore, *Smc5* cKO alters brain FC and may predispose patients to increased developmental delays (Figure 7). Research and therapies have focused on the reduction of hypoxic events for patients with severe CHD. However, this may be insufficient if our goal is to reduce the total risk for NDD in patients with CHD. Further research should investigate whether genetic variants can help stratify patients at greater risk for NDD and identify patients who may benefit most from gene therapies.

## 4. Materials and Methods

### 4.1. Editorial Compliance and Ethical Considerations

All the human subject research was approved by the Yale University Institutional Review Board, and all the animal studies were approved by the Institutional Animal Care and Use Committee of Johns Hopkins University and Yale University. Written informed consent was obtained from all the family members for the sharing of de-identified research results for publication.

### 4.2. Patient Recruitment and Whole Exome Sequencing

An obstetrician referred the patient for trio whole exome sequencing (patient and parents) based on results obtained from a prenatal cardiac ultrasound. The saliva for DNA extraction was collected from the trio with the DNA Genotek kit (DNA Genotek, Ottawa, ON, Canada) after delivery. The genomic DNA of the trio was prepared from saliva using standard procedures. The whole exome was captured using the xGen target kit (Integrated DNA Technologies, Coralville, IA, USA), and 99 base paired-end sequencing on the Illumina platform (HiSeq 4000, Illumina, San Diego, CA, USA) was performed under a research protocol at the Yale Center for Genome Analysis. The sequence reads were converted to FASTQ format and were aligned to the reference human genome (hg19). GATK best practices were applied to identify genetic variants [86], and variants were annotated by ANNOVAR V3.1.2 [87]. The proband’s and their parents’ DNA was sequenced to a mean depth of 56–68× independent reads per targeted base, with at least 20× independent reads in over 92% of targeted bases.

We filtered exonic or splice-site rare variants (minor allele frequency ≤ 0.01 across all the samples in 1000 Genomes, NHLBI-EVS, gnomAD all or subpopulations, and our institutional database) that exhibited high-quality sequence reads. We searched for rare recessive or de novo variants in the patient. All the recorded variants were then visualized and verified manually by Integrative Genomics Viewer (IGV) V2.8.13 (https://igv.org/doc/desktop/, accessed on 1 December 2018 and 27 May 2021). The only novel de novo variant in the proband is NC_000009.11:g.72930398delT c.1710del (p.F570fs*7) variant in *SMC5*, which appeared 21 times from the total of 40 reads in the proband, whereas this variant was not detected in either parent with an average of ~30 reads. No homozygous or compound heterozygous rare non-synonymous variants (<0.05%) with higher CADD scores (>20) were identified from the patient.

### 4.3. Echocardiography

The fetal echocardiography was obtained at 19 and 23 weeks of gestation by transabdominal ultrasound of the patient’s mother without fetal or maternal sedation. The postnatal echocardiography was obtained at 28 days of life after surgical repair. The fetal and postnatal echocardiography was obtained and analyzed via the Epiq 7C Ultrasound system (Philips, Amsterdam, The Netherlands). Echocardiography analysis software ApolloLX V 7.0.1 (LUMEDX, Oakland, CA, USA) was used to measure and quantify the size of cardiac structures. The z-scores were calculated based on the Boston Children’s Hospital z-score calculator [88].

### 4.4. X. tropicalis Husbandry and Habitat

The *X. tropicalis* animals were housed and cared for in our aquatic facility following Yale University Institutional Animal Care and Use Committee and IACUC protocols. The embryos were produced by in vitro fertilization and raised to appropriate stages in 1/9× Modified Ringer’s as previously described [89].

### 4.5. X. tropicalis Genome Editing and Genotyping

In vitro fertilization and embryo microinjection were performed as previously described [90]. Non-overlapping sgRNAs targeting *smc5* exons 1 and 4 were used for CRISPR-Cas9 mediated gene knockout. sgRNA oligonucleotides were designed using CRISPRscan sgRNA selection tool (https://www.crisprscan.org/, accessed on 21 August 2018) [91] and version 9.1 of *X. tropicalis* genome to target the sequences given in Supplementary Table S2. sgRNA targets were selected if they contained a low risk for off-target effects with a cutting frequency determination (CFD) score of <10 [92], and a cutting efficiency score of >50 [91]. sgRNA was transcribed using the NEB Engen sgRNA Synthesis Kit (New England Biolabs, Ipswich, MA, USA) and purified using the Zymo Quick-RNA Miniprep kit (Zymo Research, Irvine, CA, USA). At the one-cell stage, *X. tropicalis* embryos were injected with 400 pg of sgRNA, 1.6 ng Cas9 protein (PNABio, Thousand Oaks, CA, USA), and 1.6 μg Alexa Fluor 488 dye (Invitrogen, Waltham, MA, USA) using a fine glass needle and Picospritzer (Parker-Hanifen, Cleveland, OH, USA). Embryos were raised in 1/9× Modified Ringer’s solution supplemented with 50 μg/mL gentamycin as previously described to the required stage of development [89].

To verify CRISPR editing accuracy and efficiency, pooled tadpole genomic DNA from 5 injected tadpoles at stage 45, as well as pooled genomic DNA from 5 uninjected stage 45 tadpoles, was extracted using the DNAEasy Blood and Tissue kit (Qiagen, Germantown, MD, USA). Genomic DNA was amplified using Platinum Taq polymerase (ThermoFisher, Waltham, MA, USA) with primers designed to amplify the region from ~250 nucleotides upstream to ~500 nucleotides downstream of the cut site. The genotyping primers used are listed in Supplementary Table S3. PCR products were purified using Monarch PCR Cleanup kit (New England Biolabs), and purified products were sequenced via Sanger sequencing, and analysis of CRISPR editing efficiency and insertion/deletion status were determined with ICE (Inference of CRISPR Edits) analysis [93].

### 4.6. Optical Coherence Tomography of X. tropicalis Heart and Brain

The brain and heart size of *X. tropicalis* embryos were measured at stage 45 in control and CRISPR-edited tadpoles using optical coherence tomography (OCT) with the Thorlabs Telesto 1325 nm spectral domain-OCT system (Thorlabs, Newton, NJ, USA), as previously described [94]. For the evaluation of brain size, tadpoles were temporarily anesthetized with a solution of 0.05% benzocaine. For the evaluation of heart size, tadpoles were evaluated without anesthesia due to the effect of benzocaine on cardiac function. All the tadpoles were placed in 1% low-melt agar cooled to 30 °C to reduce movement during imaging. To capture heart length at end diastolic and end systolic time points, cardiac two-dimensional (2D) cross-sectional movies from a ventral view of the tadpole were obtained at high-speed mode-91 kHz (91,000 A-scan per second) with 91 dB sensitivity to capture the complete cardiac cycle, which varies between 110–140 beats/min. The maximal ventricle diastole and systole diameter were measured with 3 separate measurements recorded from wall-to-wall. The end-diastolic diameter (EDD) and end-systolic diameter (ESD) were recorded as the average of these three measurements. To capture the brain size, a dorsal view of the tadpole was captured with cross sections spanning the length of the tadpole head to create a three-dimensional (3D) reconstruction. The brain length was then measured from the most anterior aspect of the cortex to the start of the spinal cord (the inflection points where the tapering of the posterior brain meets the linear spinal cord). The brain length was measured with 3 separate measurements and was recorded as the average of these three measurements.

### 4.7. Whole Mount In Situ Hybridization of X. tropicalis Embryos

A digoxigenin-labeled antisense probe for *pax6* was in vitro transcribed using the T7 High Yield RNA Synthesis Kit (New England Biolabs). The embryos were collected at the desired stages, fixed in MEMFA formaldehyde solution, and dehydrated in 100% ethanol. Whole-mount in situ hybridization of digoxigenin-labeled antisense probes was performed overnight, the labeled embryos were then washed, and incubated with Roche anti-digoxigenin-AP Fab fragments (Roche Diagnostics 11093274910, Basel, Switzerland), and a signal was detected using BM-purple (Roche 11442074001), as previously described [95]. A Canon EOS 5D digital camera mounted on a Zeiss Discovery V8 stereomicroscope (Oberkochen, Germany) was used for imaging.

### 4.8. mESC Differentiation into Cardiomyocytes and Their Characterization

The mESC Smc5-1 (cKO; *Smc5* flox/del, Cre-ERT2) and mESC Smc5-3 (control; *Smc5* wt/flox, Cre-ERT2) line maintenance and characterization were previously described [30]. For cardiac differentiation, the mESCs were plated as single cells at ~8000 cells/cm^2^ onto gelatinized plates in differentiation medium (RPMI (Corning, 15-040, Corning, NY, USA), supplemented with 300 mg/L L-glutamine, (Sigma, G8540, St. Louis, MO, USA), 0.5 mg/mL BSA (Sigma, A1470), 0.21 mg/mL L-Ascorbic acid 2-phosphate sesquimagnesium salt hydrate (Sigma, A8960), 1× ITS (Corning 25-800-CR) and 5% FBS (HyClone, Logan, UT, USA)) [33]. 10 ng/mL Activin A (Proteintech, HZ-1138, Rosemont, IL, USA) was added on days 0–2, 6 µM CHIR 99021 (Cayman Chemical, 13122, Ann Arbor, MI, USA) was added on days 2–4, and 1 µM XAV939 (Cayman Chemical, 13596) on days 4–6 [33,34]. Starting on day 6, cells were maintained in differentiation medium, with medium change every other day. Tamoxifen (Cayman Chemical, 14854) was added at 0.2 µM starting day 6, 8, or 10 and kept in the medium until the end of the experiments.

Immunocytochemistry for sarcomere markers α-actinin (Abclonal, Woburn, MA, USA, A3718, 1:1000 dilution) and cardiac troponin T (Abclonal, A4914, 1:1000 dilution) was performed as described [96]. Cell imaging was performed using a Keyence BZ-X800 fluorescence microscope (Osaka, Japan). Images were analyzed and processed using the Keyence BZ-X800 Viewer (V01.03.00.01) and Analyzer software (V1.1.2.4). Photoshop V13.0 (Adobe, San Jose, CA, USA) was used to prepare figure images. The cell growth area calculation was performed using ImageJ V1.54f (National Institutes of Health, MD, USA) [97].

Spontaneous cardiomyocyte contractility was recorded using a Teledyne Lumenera Infinity 8 monochrome CMOS camera (Lumenera Corporation, Ottawa, ON, Canada) and Infinity Analyze microscopy software V6.5.6 (Lumenera Corporation, Ottawa, ON, Canada). Videos were processed using MiniTool MovieMaker 7.0.1 (Vancouver, BC, Canada, https://moviemaker.minitool.com/, accessed on 19 September 2023) and analyzed using Myocyter v.1.5 [98]. The data were plotted in GraphPad Prism 10.

### 4.9. Mouse Husbandry and Genotyping

All the mice were bred at Johns Hopkins University (JHU, Baltimore, MD) in accordance with the National Institutes of Health and U.S. Department of Agriculture criteria, and protocols for their care and use were approved by the Institutional Animal Care and Use Committee (IACUC) of JHU. Mice of the following strains were used: C57BL/6J (B6/J), stock number 000664 (Jackson Laboratory (JAX), Bar Harbor, ME, USA); B6.Cg-Tg(*Nes*-Cre)1Kln/J (*Nestin*-Cre), stock number 003771 (JAX). Mice harboring *Smc5* with a floxed exon 4 (designated *Smc5* flox) and deleted exon 4 (designated *Smc5*del) have been previously described [20,30]. These mice were bred to obtain the following *Smc5* cKO genotype: *Smc5* flox/flox, *Nestin*-Cre tg/0. Mice from both sexes were included in the study. Genotypes not resulting in the homozygous gene KO were used as controls. PCR genotyping was performed using AccuStart II PCR SuperMix (Quanta BioSciences, Beverly, MA, USA), as documented previously [20]. Mice were exposed to a 14-h light/10-h dark-light cycle facility at the breeding and storage facility before their transfer to Yale.

### 4.10. MRI of Mouse Brain

All the procedures were performed in accordance with the Yale Institutional Animal Care and Use Committee and are consistent with the National Institute of Health Guide for the Care and Use of Laboratory Animals. On an 11.7T preclinical Bruker magnet (Bruker, Billerica, MA, USA), functional and structural MRI data were collected from 13 adult mice (age 18–22 weeks, body weight 34 ± 3 g, mean ± standard deviation). For data acquisition, and all subsequent analyses, the experimenter was blinded to genotype status (n = 7 *Smc5* flox/flox, *Nes*-Cre tg/0, herein called ‘*Smc5* cKO’, or n = *5 Smc5* flox/flox, herein called ‘control’). One mouse of the original 13 mice in the study was excluded before scanning due to an inability to verify the genotype. While at Yale before scanning, mice were housed in a 12-h light/dark cycle facility, and food and water were available ad libitum. All the mice completed the scan protocol with no signs of distress. To ensure blinded data acquisition, no overt physical differences were observed which could suggest genotype status. The structural and functional data were obtained using standard sequences and analyzed using established protocols and software.

### 4.11. Obtaining fMRI of Mouse Brains

Anesthesia was induced using 3% isoflurane using a mixture of O_2_ and medical air (1:3). Once unresponsive, mice were transferred to an in-house built bed with an incisor bar and padded vice which cradles the sides of the head to reduce motion. For the MR signal reception, we used a custom in-house-built saddle coil. During imaging, isoflurane was reduced to 0.5–0.75%. The body temperature was continuously monitored using a rectal probe and maintained using a circulating water bath.

We collected the fMRI data during rest (no stimulation). The BOLD fMRI data were collected using a gradient-echo, echo-planar-imaging (GE-EPI) sequence. Image resolution was 0.275 × 0.275 × 0.275 mm^3^, TR = 1.8 s, and TE = 9.1 ms. We collected 35 slices which yields close to whole-brain coverage. Each functional imaging run consisted of 334 repetitions (~10 min). In total, from each mouse, we acquired 30 min of resting-fMRI data.

In addition to the fMRI data, we collected three structural scans. First, we obtained a high in-plane resolution multi-spin-multi-echo (MSME) image within the fMRI field-of-view (FoV). In 7 min, 20 s, using a TR/TE of 2500/20 ms, we obtained 35 slices (0.275 mm thick) with an in-plane resolution of 0.1 × 0.1 mm^2^ (two averages). The slice prescription of these images matched those of the fMRI data (i.e., they were of the same anatomy). Next, we obtained an isotropic 3D anatomical image of the whole brain. Using an MSME imaging sequence, in 8 min and 4 s, using a TR/TE of 5500/15 ms, we obtained a 0.2 × 0.2 × 0.2 mm^3^ resolution image (two averages). Each MSME acquisition was repeated interleaved between fMRI data acquisitions (for a total of four averages each). This allowed the gradients to cool and has been shown to improve the reliability of connectivity measurements from fMRI data [99]. In post-processing, MSME high in-plane resolution (fMRI FOV) and whole-brain isotropic images, were each concatenated, motion corrected, and averaged to create one of each image. This was done in BioImageSuite (V1.1.0b8, Yale University, CT, USA, www.bioimagesuite.org, accessed on 19 November 2019) [100]. Finally, we obtained an MR-angiogram. Using a fast-low-angle-shot (FLASH) time-of-flight (TOF) imaging sequence, in 24 min, using a TR/TE of 130/4 ms, we obtained a 0.075 × 0.075 × 0.075 mm^3^ resolution image of the vasculature with a FoV of 1.5 × 1.1 × 0.7 cm^3^ which contained the whole-brain.

### 4.12. Analysis of Mouse MRI Data

All the MRI data processing was conducted in Analysis of Functional NeuroImages (AFNI, V19.2.21, National Institutes of Health, MD, USA), MATLAB (V9.8, R2020a, Natick, MA, USA), and BIS (V1.1.0b8, Yale University, CT, USA, www.bioimagesuite.org, accessed on 19 November 2019). Functional MRI data were motion corrected (6-parameters) (AFNI, 3dvolreg), masked to isolate brain tissue (BIS), filtered (0.008–0.2 Hz) (MATLAB, Butterworth), the global signal regressed (MATLAB, detrend), and blurred to a full-width-half-maximum (FWHM) of 0.3mm (MATLAB, smooth-gaussian).

Following brain segmentation, whole-brain MSME images were registered to a common space using a non-linear, non-rigid registration method based on normalized mutual information (BIS) [101]. The common space to which the 3D MSME image from each mouse was registered was created from an independent group of n = 162 mouse MRI data sets we have collected for a separate study. These data have been registered to the Allen Atlas [44]. Both the reference space and the registered Allen Atlas are publicly available (BIS, V1.1.0b8, Yale University, CT, USA, www.bioimagesuite.org, accessed on 19 November 2019) [100]. Next, for each mouse, we used anatomical landmarks to register the high in-plane resolution MSME image, which matches the EPI FoV, to the isotropic resolution whole-brain MSME image, and the EPI image to which the time series was registered during motion correction (BIS). Together, these transformations (reference space → high in-plane resolution MSME → isotropic MSME → EPI) allowed us to move the Allen Atlas into the native fMRI space of each mouse for FC measurements. Moving the Allen Atlas into the native space of the isotropic MSME image of each mouse enabled us to measure the volume of each brain region. Transformations are concatenated before they are applied. Supplementary Figure S2 depicts an overview of these steps. Similarity between difference matrices was computed using Pearson’s correlation.

The MR angiography data were used to measure the intra-luminal volume of the major blood vessels in the brain. These data were segmented using signal intensity thresholding (BIS).

### 4.13. Statistical Analysis

For the mouse MRI data, the *p*-values from groupwise comparisons were generated using MATLAB *ttest2* (V9.8, R2020a, Natick, MA, USA) unless otherwise noted. For the frog data and mouse cardiomyocytes, the statistical analyses were performed using GraphPad Prism 9 or 10 and specified in the relevant figure legend. Significance was indicated by a *p*-value < 0.5.

## Data Availability

The data that support the findings of this study are available from the corresponding authors upon reasonable request.

## Supplementary Materials

The following supporting information can be downloaded at: https://www.mdpi.com/article/10.3390/ijms25010430/s1.

## Abbreviations

CHD	congenital heart disease
NDD	neurodevelopmental disability; cKO: conditional knockout
HLHS	hypoplastic left heart syndrome
LOF	loss of function
FC	functional connectivity
BOLD	blood oxygen level dependent
fMRI	functional magnetic resonance imaging
OCT	optical coherence tomography
SMC	structural maintenance of chromosomes
SLF	SMC5/6 localization factor
mESC	mouse embryonic stem cell
