# Supplementary material for: SMC5 Plays Independent Roles in Congenital Heart Disease and Neurodevelopmental Disability
Source: Int J Mol Sci. 2023 Dec 28;25(1):430. doi: 10.3390/ijms25010430 (PMC10779392; doi:10.3390/ijms25010430)
Supplement: Supplementary file 1 [file ijms-25-00430-s001.zip › Supplementary Tables and Figures.pdf]

Supplemental Table S1. *De novo*, homozygous recessive, and compound heterozygous variants in proband with hypoplastic left heart syndrome.

Variant type	Position	Gene	Gene Name	Variant	MAF
De novo	3:127292472_G>A	*TPRA1*	transmembrane protein adipocyte associated 1	g.R284*	0.0000122
De novo	9:72930395_AT>A	*SMC5*	structural maintenance of chromosomes 5	g.F570fs*7	0
De novo	X:142795437_C>T	*SPANXN2*	SPANX family member N2	g.V81I	0.0000224
Homozygous recessive	7:64168429_G>A	*ZNF107*	zinc finger protein 107	g.E583K	0.0036
Homozygous recessive	12:80199958_C>G	*PPP1R12A*	protein phosphatase 1 regulatory subunit 12A	g.G604A	0.0048
Homozygous recessive	16:84353055_C>T	*WFDC1*	WAP four-disulfide core domain 1	g.T147M	0.0002
Compound heterozygous	8:145163540_G>A	*WDR97*	WD repeat domain 97	g.V191M	0.0045
Compound heterozygous	8:145166390_G>A	*WDR97*	WD repeat domain 97	c.2487+1G>A	0.0026
Compound heterozygous	9:120474891_T>C	*TLR4*	toll like receptor 4	g.I122T	0.0002
Compound heterozygous	9:120475787_G>A	*TLR4*	toll like receptor 4	g.V421I	0
Compound heterozygous	10:46965017_C>T	*SYT15*	synaptotagmin 15	g.D310N	0.0042
Compound heterozygous	10:46968674_ AATCTC>A	*SYT15*	synaptotagmin 15	p.R86fs	0.0028

Variant position listed using chromosome number:base number format. MAF: minor allele frequency, *:stop codon, fs: frameshift, fs*7: frameshift of 7 bases creating stop codon.

Supplemental Table S2. Exon targeted, and sgRNA-specific spacer sequence used for CRISPR-Cas9 mediated gene editing in *Xenopus tropicalis*.

Targeting site	Spacer sequence
smc5 A (exon 1)	5'-GGGAGUCUGUACCAGCGAAG-3'
smc5 B (exon 4)	5'-GGUGGAGAGAAAGCGACCCU-3'
tpra1 A (exon 2)	5'-GGGCUGCACGCUUGUCACAA-3'
tpra1 B (exon 5)	5'-GGGCUCAGTGUGGUGAUCCU-3'
tpra1 C (exon 9)	5'-GGUGCCCUGAACCUGGUCCA-3'
tpra1 D (exon 5)	5'-GGUUUCCCAAAGGACCUGAA-3'

Supplemental Table S3. Primers used for PCR amplification of *X. tropicalis* genomic DNA.

**Target**	**Forward Primer'(5'-3')**	**Reverse Primer'(5'-3')**
Smc5 exon 1	AGATAGGAACGCCAATACTTTCGT	AAGCATCCAAAGAGACTCACGT
Smc5 exon 4	ATGGTATATTTGCCACTCCTGTGT	AATCAGAGGTAGTGTGTTGTCTAC

Supplementary Figure S1

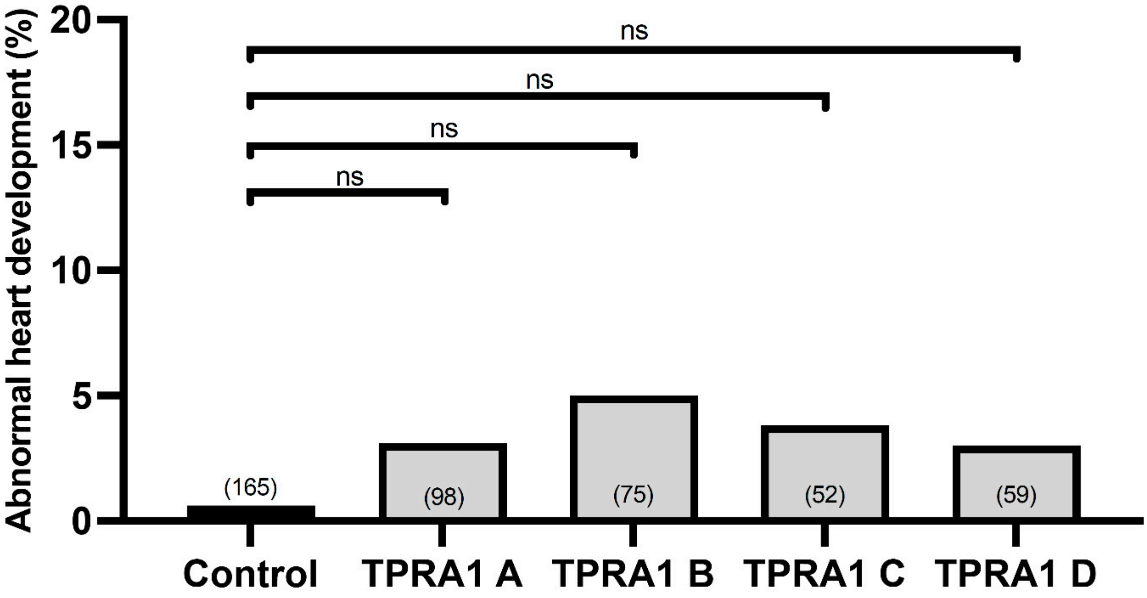

Supplementary Figure S1. *tpra1* knockout in *Xenopus tropicalis* is not associated with cardiac defects. CRISPR-Cas9 mediated knockout of *tpra1* using sgRNAs targeting using 4 separate non-overlapping target sites (Supplemental Table S2) does not cause notable developmental cardiac defects. ns: nonsignificant, numbers in parentheses indicated n values.

Supplementary Figure S2

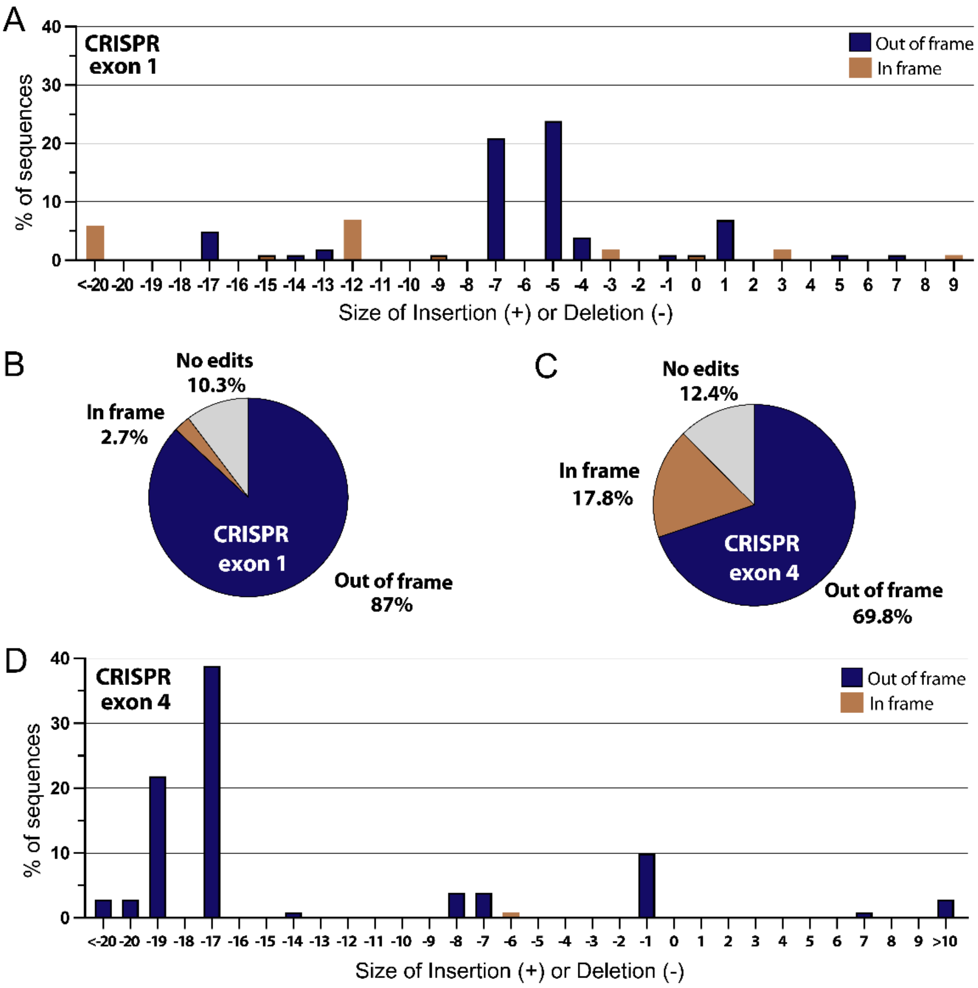

Supplementary Figure S2. CRISPR-Cas9 complexes targeting *smc5* cause insertion/deletions in *X. tropicalis* genome (**A-D**) Bar graphs show frequency and size of insertion or deletion (indel) in *smc5* induced by CRISPR-Cas9 activity. Pie graphs show type and frequency of indel caused by CRISPR-Cas9. Indel size in multiples of 3 correspond to in frame indels (brown), and samples without indels considered to have no edits. Indels of all other sizes considered out of frame edits (blue). DNA samples without edits not represented on bar graph. (**A**) CRISPR-exon 1 indel size and frequency, (**B**) CRISPR-exon 1 edit type and frequency, (**C**) CRISPR-exon 4 edit type and frequency, (**D**) CRISPR-exon 4 indel size and frequency. n=5 for all experiments.

Supplementary Figure S3

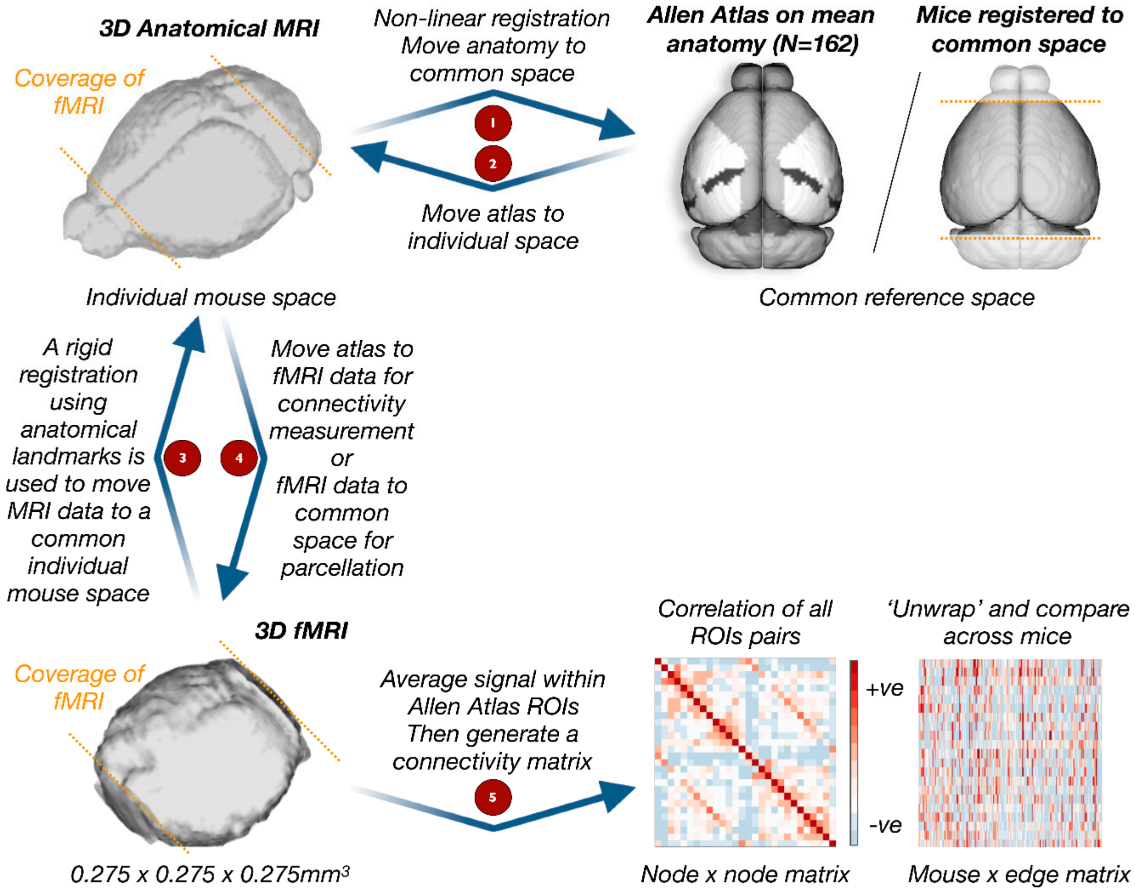

Supplementary Figure S3. Vessel volume was correlated with total brain volume Anatomical data from individual mice are registered to a common space. We use a reference space created from the nonlinear registration, and subsequent averaging, of n=162 mice. This reference space has been registered to an MRI compatible version of the Allen Atlas. This allows us to estimate the volume of different regions of interest (ROI) from the Allen Atlas for each mouse and to impose the Allen Atlas onto the fMRI data for functional connectivity measurements.

Supplementary Figure S4

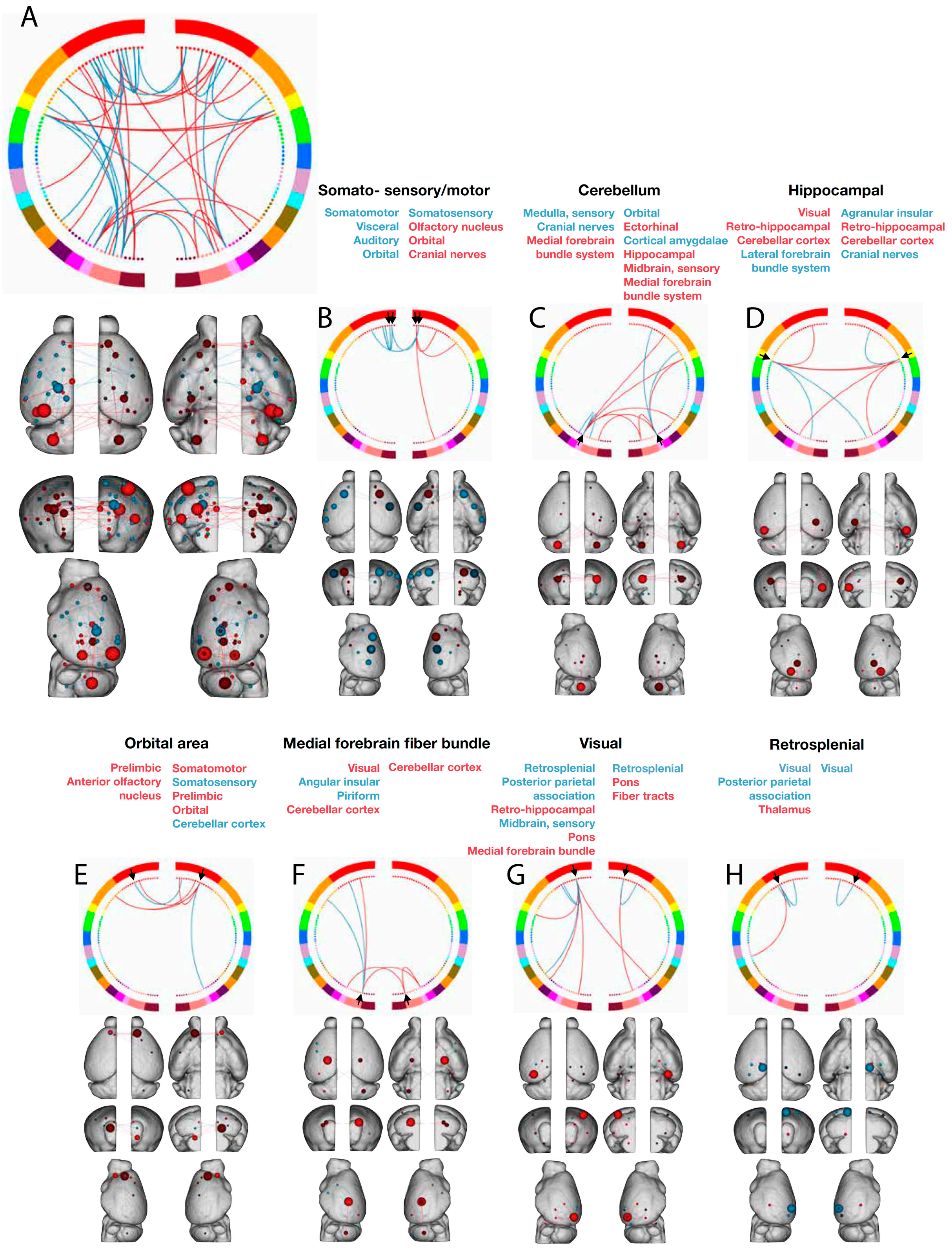

Supplementary Figure S4. Connectivity matrix can be interrogated to assess mouse functional connectivity between and within groups. (**A**) Edge (region to region correlation) strengths averaged for control (salmon) and cKO (sky blue) mice. There are no group differences the distribution of edge strengths between groups, inter or intra-hemisphere. Kurtosis and skewness were found to show no groupwise differences when compared both inter- and intra-hemisphere. (**B**) Connectivity between each brain region is shown, compared interhemisphere and intrahemisphere. The connectivity patterns of control mice are shown in the top right half of the matrix, and those of cKO mice are shown in the bottom left half of the matrix. (**C**) To identify differences between control and cKO connectivity patterns, difference matrices are computed between all pairs of mice. Both within the control and cKO groups, as well as comparisons between groups, all show a weak, but significant, correlation between distinguishing connectivity profiles. (**D**) We average the difference matrices, threshold the edges, and binarize to generate two networks which distinguish control from cKO mice. The two networks consist of either edges that show higher connectivity strength in control than cKO mice (Positive network), or lower connectivity strength in control than cKO mice (Negative network). n=12 for all experiments.

Supplementary Figure S5

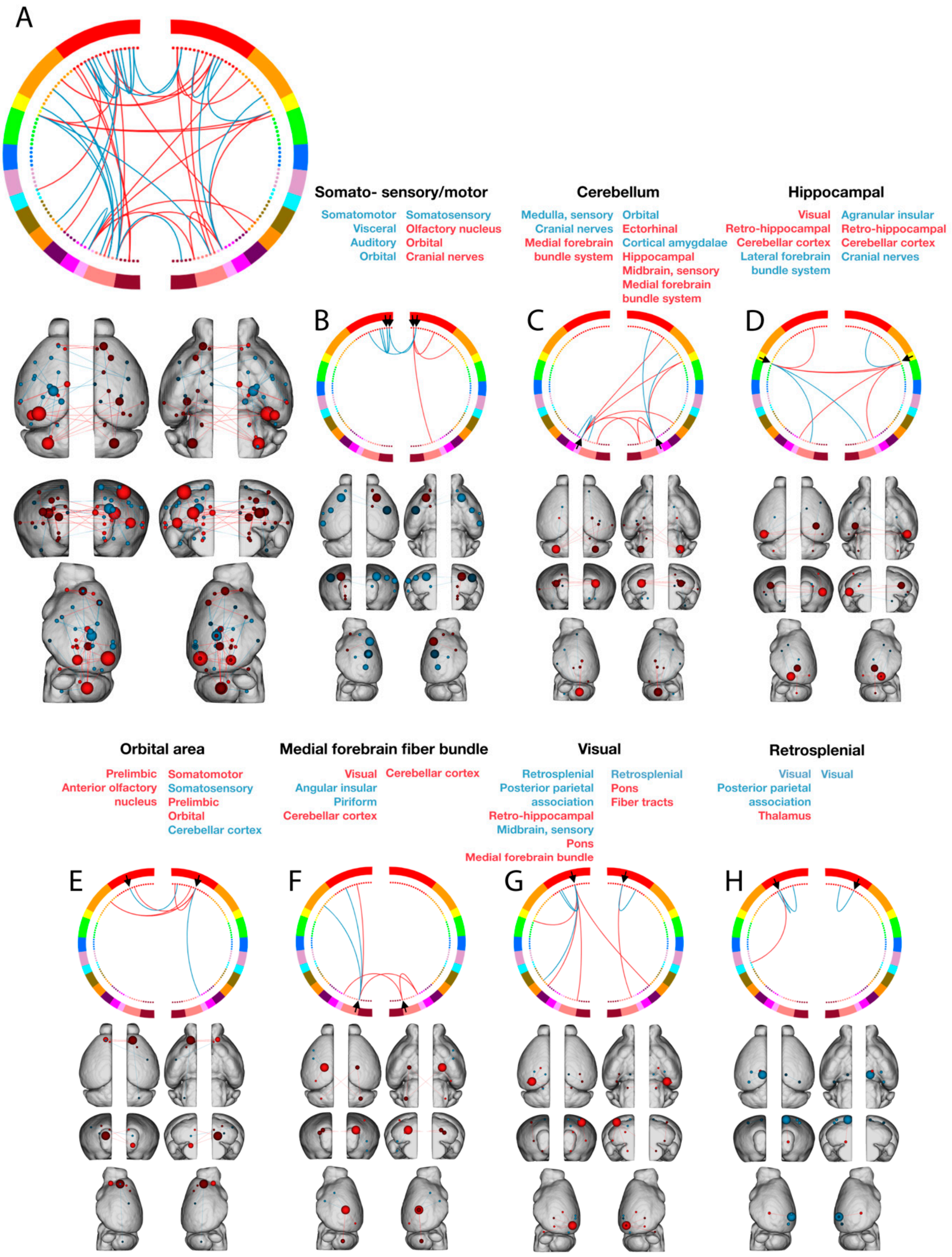

Supplementary Figure S5. *Smc5* cKO disrupts functional connectivity in all brain regions interrogated. Connectivity maps and corresponding mouse brain representations with respective regions of interest (ROIs) described by black text atop of each section. Lines shown between the ROI and another region represent a significant change in connectivity compared to control mice. Red lines represent increased synchrony and blue lines represent decreased synchrony. List of brain regions above connectivity map summarize regions that show significant differences in connectivity. Volumetric representation of mouse brain from each visual angle, with topmost images showing dorsal (left) and ventral (right) aspects, middle images showing rostral (left) and caudal (right) aspects, and bottom set of images showing left lateral (left) and right lateral (right) aspects of the mouse brain. **(A)** Combined connectivity changes across the brain, with all ROIs shown. Additional connectivity maps with ROIs in somatosensory and somatomotor regions **(B),** cerebellum **(C),** Hippocampus **(D),** Orbital area **(E)**, Medial forebrain bundle **(F)**, Visual cortex **(G)**, and Retrosplenial region **(H)**. n=12 for all experiments.

Supplemental Movie S1. Example movie of beating TAM-treated control cardiomyocytes used for data presented in Figure 2H.

Supplemental Movie S2. Example movie of beating untreated (Unt) *Smc5* cKO cardiomyocytes used for data presented in Figure 2H.

Supplemental Movie S3. Example movie of fast beating TAM-treated *Smc5* cKO cardiomyocytes used for data presented in Figure 2H.

Supplemental Movie S4. Example movie of slow beating TAM-treated *Smc5* cKO cardiomyocytes used for data presented in Figure 2H.

Supplemental Movie S5. Example movie of slow and fast beating TAM-treated *Smc5* cKO cardiomyocytes used for data presented in Figure 2H.

Supplemental Movie S6. Example movie of variability of beating rhythm in TAM-treated *Smc5* cKO cardiomyocytes used for data presented in Figure 2J.
